# Urban-rural differences in immune responses to mycobacterial and tetanus vaccine antigens in a tropical setting: A role for helminths?

**DOI:** 10.1016/j.parint.2020.102132

**Published:** 2020-10

**Authors:** Joyce Kabagenyi, Agnes Natukunda, Jacent Nassuuna, Richard E. Sanya, Margaret Nampijja, Emily L. Webb, Alison M. Elliott, Gyaviira Nkurunungi

**Affiliations:** a Immunomodulation and Vaccines Programme, MRC/UVRI and LSHTM Uganda Research Unit, Entebbe, Uganda; b College of Health Sciences, Makerere University, Kampala, Uganda; c MRC Tropical Epidemiology Group, Department of Infectious Disease Epidemiology, London School of Hygiene and Tropical Medicine, London, United Kingdom; d Department of Clinical Research, London School of Hygiene and Tropical Medicine, London, United Kingdom

**Keywords:** Vaccine, Tetanus toxoid, Purified protein derivative, Urban-rural, Cytokine, Antibody

## Abstract

Several vaccines elicit lower efficacy or impaired immune responses in rural compared to urban settings, and in tropical low-income countries compared to high-income countries. An unresolved hypothesis is that immunomodulation by parasitic infections such as helminths (prevalent in rural tropical settings) contributes to suppression of vaccine responses. Among 1–17-year-old Ugandan residents of rural *Schistosoma mansoni* (*Sm*)-endemic islands and proximate urban communities with lower helminth exposure, we assessed plasma antibody and whole blood assay cytokine responses to tetanus toxoid (TT) and purified protein derivative of *Mycobacterium tuberculosis* (PPD). These were taken to represent recall responses to tetanus and BCG vaccination in infancy. PPD-specific responses are additionally induced by tuberculous and non-tuberculous mycobacterial exposure. Urban-rural comparisons showed that PPD-specific IFN-γ and IL-13 and TT-specific IL-13 and IgG concentrations were lower in the rural setting, but that PPD-specific IgE concentrations were higher. Among rural participants, *Sm* infection was inversely associated with PPD-specific IFN-γ, while nematode infection was positively associated with PPD-specific IgG. Among urban participants, *Sm* infection was positively associated with PPD-specific responses but inversely associated with TT-specific responses, while nematode infection was inversely associated with TT-specific IgG and IgG4, but no associations were observed with PPD-specific responses. Despite these associations, for the urban-rural comparisons there were no notable changes in test statistics after adjusting for current helminth infections, suggesting that helminths were not the sole explanation for the urban-rural differences observed. Helminths likely work in concert with other environmental exposures and operational factors to influence vaccine response.

## Highlights

• Vaccine (BCG, tetanus)-specific immune responses differ by urban/rural setting.
• Associations between helminths and vaccine-specific response also differ by setting.
• Urban-rural differences are not fully explained by helminth infection prevalence.
• Helminths likely work in concert with other factors to influence vaccine response.

## Graphical abstract

**Figure:**
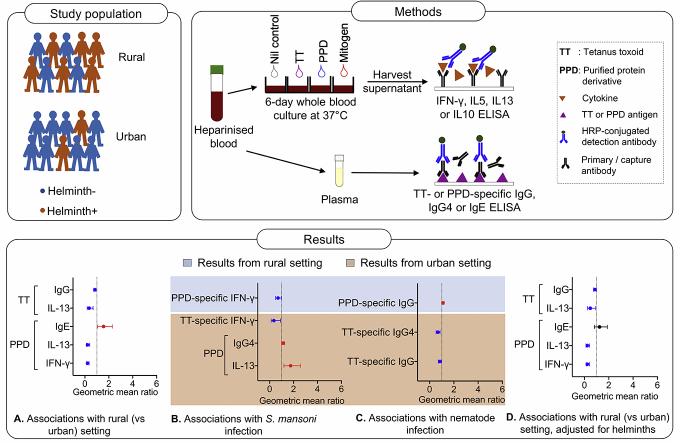

## 1 Introduction

Effective vaccines play a major role in control of infectious diseases; however, several licensed [[1], [2], [3], [4]] and candidate [[5], [6], [7]] vaccines are less efficacious, and vaccine-specific immune responses impaired, in tropical compared to higher latitudes. This phenomenon is best documented for Bacillus Calmette-Guérin (BCG) [2,3]; however, similar trends have been observed for other vaccines. For example, levels of neutralising antibodies against yellow fever vaccine are lower, and wane faster, in Uganda compared to Switzerland [1]. Responses to novel viral-vectored vaccines are lower among African individuals compared to their United Kingdom counterparts [6,7]. Some enteric vaccines also show variable efficacy between low- and high-income countries [8,9]. In the tropics, rural settings appear more affected [[10], [11], [12], [13]]. For example, influenza and tetanus vaccine responses have been shown to be lower in rural compared to urban Gabon [12,13].

Why several vaccines are less efficacious in tropical low-income countries compared to high-income countries, and in rural versus urban settings, is incompletely understood. BCG vaccine efficacy in migrant and native populations in England is comparable [14], hence genetic differences may not fully explain population differences in vaccine response. Prior exposure to the target, or related, organisms, may mask the benefit of the vaccine [15]: exposure to non-tuberculous mycobacteria pre- [16] and post-BCG immunisation [15] was shown to modify protection induced by BCG in mice. However, pre-immunisation exposure cannot explain observations for vaccines against rare pathogens, such as Ebola [7].

Another long-held hypothesis is that helminth infections, which are highly prevalent in rural tropical settings, modulate vaccine responses by suppressing the Th1 responses necessary for protection against several pathogens targeted by vaccines [17,18]. In animal models, helminths generally impair priming and accelerate waning of vaccine responses, but effects vary with helminth species and vaccine type [19]. In humans, treating geohelminths has been shown to improve responses to BCG [20,21] and oral cholera vaccine [22], and a recent study in Uganda suggested that treatment of schistosomiasis improves the measles-booster response in children [23].

We surveyed helminth-endemic Lake Victoria islands of Koome, Uganda [[24], [25], [26], [27]] and proximate mainland urban communities with lower helminth exposure [28] as part of a set of studies on helminths and allergy-related outcomes. Vaccine responses were measured as a secondary outcome in these surveys. Logistics did not permit us to administer the corresponding vaccines; however, we anticipated that assessment of vaccine-specific immune responses among survey participants would contribute to planning of further studies designed specifically to examine effects of environmental and parasite exposures on vaccine efficacy. Here, we present results from an urban-rural comparison of immune responses to mycobacterial and tetanus vaccine antigens, and explore the hypothesis that differences, if any, are attributable to differential helminth exposure intensity between the two settings. Understanding drivers of urban-rural differences in vaccine response may be key to maximising the effectiveness not only of licensed, but also of candidate vaccines, in the tropics.

## 2 Methods

### 2.1 Study settings and procedures

Samples for the present analysis were obtained from 1 to 17-year-old participants of two cross-sectional surveys conducted in Uganda, one in 26 helminth-endemic rural fishing villages of the Lake Victoria Koome islands and the other in Entebbe municipality, an urban setting with lower helminth infection exposure, located on the northern shores of Lake Victoria, approximately 35 km from Koome.

The “rural survey” was the outcomes survey (September 2015 – August 2016) following three years of the Lake Victoria Island Intervention Study on Worms and Allergy-related diseases (LaVIISWA; ISRCTN47196031), a cluster-randomised trial of community-based standard versus intensive anthelminthic treatment, described previously [24,26]. The “urban survey” (September 2016 – September 2017) was designed purposely to collect data for comparison with the rural survey [28]. Urban survey participants were not randomised to standard versus intensive anthelminthic intervention; however, all other procedures were designed to be equivalent between the two surveys.

The primary outcomes for both surveys were allergy-related [26,28]. This paper reports results for the pre-specified secondary outcomes of immune responses to tetanus and BCG vaccination. Both vaccines had been administered by the Uganda National Expanded Programme on Immunisation (UNEPI) in infancy with good reported coverage [29]. The Ministry of Health (Uganda) also recommends tetanus booster immunisation in adolescence (but coverage is variable) and during pregnancy [30]. For logistical reasons it was not possible for the research team to administer the vaccines during this project.

The surveys collected socio-demographic and clinical data from consenting / assenting individuals. Data included history of immunisations, including with BCG and tetanus in infancy, obtained from health cards where available; in the absence of health cards, parent/caregiver's recall, another person's recall or a participant's own response was recorded. Stool and blood were also obtained for laboratory analyses.

Both surveys were approved by research ethics committees of Uganda Virus Research Institute (refs: GC/127/12/05/03 and GC/127/16/02/547) and London School of Hygiene and Tropical Medicine (refs: 6187 and 10709), and the Uganda National Council for Science and Technology (refs: HS1183 and HS2036).

### 2.2 Laboratory methods

To assess infection with *Schistosoma mansoni* (*Sm*) and other intestinal helminths, one stool sample per participant (two slides, different technologists) was examined using the Kato-Katz (KK) technique [31]. The remaining sample was suspended in 70% ethanol and stored at −80 °C for later determination of *Sm, Necator americanus* and *Strongyloides stercoralis* infections using multiplex real-time PCR [32,33].

Outcomes for the current analysis were cytokine and antibody responses to tetanus toxoid (TT) and purified protein derivative (PPD), used here to denote responses to tetanus and BCG vaccination, respectively. Additionally, PPD-specific responses are elicited by exposure to tuberculous and non-tuberculous mycobacteria.

We assessed stimulated interferon (IFN)-γ (T helper [Th]1), interleukin (IL)-5, IL-13 (both Th2) and IL-10 (regulatory) production in a six-day whole blood assay (previously described [34]), among all urban and rural survey participants from whom we obtained a sufficient blood sample both for this assay, and related cellular assays (not reported here). Briefly, we diluted heparinised blood to a final concentration of 1-in-4 using RPMI 1640 medium supplemented with glutamine, streptomycin, HEPES buffer and penicillin (all from Life technologies, UK) and cultured it (at 37 °C, 5% CO_2_) in 96-well, round-bottomed plates (Corning, USA) with PPD (10 μg/ml) or TT (12 Lf/ml) [both from Statens Serum Institut, Denmark] or phytohaemagglutinin (PHA, 10 μg/ml; Sigma, UK), or left it unstimulated. On culture day six, supernatants were harvested and stored at −80 °C. Supernatants were later thawed and analysed for cytokine levels using commercial ELISA kits (Becton Dickinson, USA). We calculated the net cytokine levels in each antigen well by deducting the concentration in the unstimulated well. Net cytokine concentrations that were negative or lower than the assay dynamic range were set to zero. For both surveys, whole blood assays were conducted using the same antigen batches and assay conditions. Each ELISA assay plate comprised samples from both surveys. All assays were conducted by the same technicians (JK, JN).

Tetanus toxoid- and PPD-specific immunoglobulin (Ig)G, IgG4 and IgE were measured in plasma using an in-house ELISA. Full details for each assay are described in this article's supplementary information. Briefly, 96-well plates were coated overnight at 4 °C with 5 μg/ml of PPD or TT and two-fold dilutions of human IgG or IgG4 or IgE standards. Plates were blocked at room temperature with 1% skimmed milk and incubated overnight at 4 °C with diluted plasma samples. Specific IgG was detected using polyclonal rabbit anti-human IgG conjugated to horseradish peroxidase. Specific IgG4 or IgE was detected using biotinylated monoclonal mouse anti-human IgG4 or IgE and a streptavidin-horseradish peroxidase conjugate. *O*-phenylenediamine was used as a substrate. Reactions were stopped with 2 M sulphuric acid. Optical density values were measured at 490 nm (reference wavelength 630 nm) using an ELISA reader. Antibody concentrations (ng/ml) were interpolated from standard curves using a five-parameter curve fit.

### 2.3 Statistical methods

Data were analysed using Stata 15.0 (College Station, TX, US). Baseline characteristics were tabulated and compared between urban and rural settings using chi-squared tests. Analyses were initially conducted on data merged from the two surveys, to assess whether PPD- and TT-specific responses differed between urban and rural settings. Thereafter, analyses were conducted separately for each survey comparing PPD- and TT-specific cytokine and antibody responses between (1) *Sm* infected and uninfected individuals, and (2) individuals infected with any nematode (*A. lumbricoides, N. americanus*, *T. trichiura*, or *S. stercoralis*) and uninfected individuals. Linear regression was used for the above analyses. All responses to PPD and TT were log_10_ (concentration + 1)-transformed for analysis and the results back-transformed to obtain geometric means (GM) and geometric mean ratios (GMR) with 95% confidence intervals (CI). To assess differences in PPD- and TT-specific responses between *Sm* infected and *Sm* uninfected subjects in the individual surveys, and between the urban and rural setting, both crude analyses and multivariable analyses adjusted for age, sex, BCG scar and place of birth were conducted. History of BCG or tetanus immunisation was not adjusted for, owing to the similarly high proportions of participants who reported and/or showed evidence of immunisation in both settings. In the combined analysis, to assess the potential role of helminth infection on differences in vaccine responses between urban and rural settings, additional adjustment for infection with *Sm* or any nematode was done and GMRs and *p* values before and after adjusting for helminths compared. Study design was accounted for in all the analyses: we used “svy” commands in Stata to allow for the non-self-weighting clustering by village in the rural survey and for clustering by sub-ward in the urban survey [28]. We used a 5% significance level for all analyses.

## 3 Results

### 3.1 Participants' characteristics

**Fig. 1:**
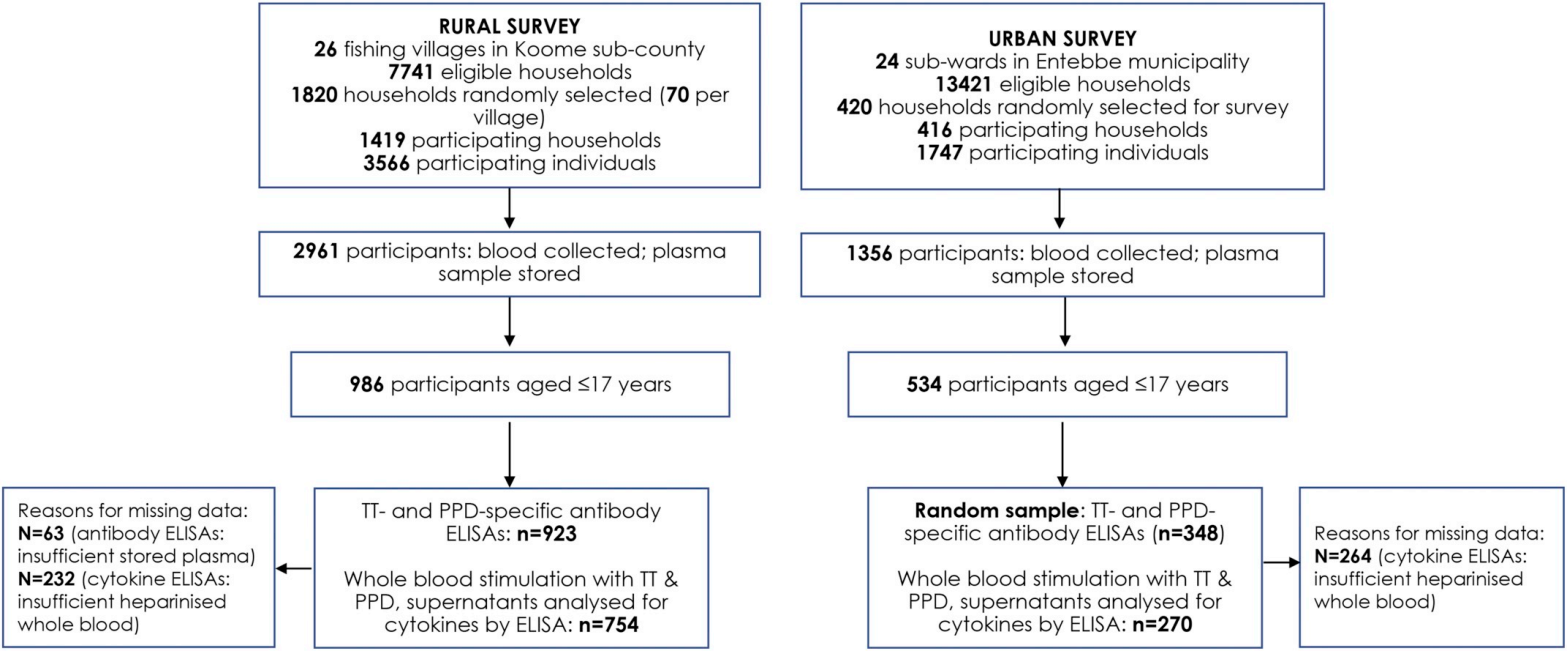
Study flowchart.

Blood samples were collected from 2961 rural survey participants, of whom 986 were aged 1–17 years: 754 of these samples were stimulated with TT and PPD (in a whole blood culture) for cytokine production. Data on plasma TT- and PPD-specific antibodies were obtained from 923 samples (Fig. 1). Blood samples were collected from 1356 urban survey participants, of whom 534 were aged 1–17 years: 270 of these samples were stimulated with TT and PPD (in a whole blood culture) for cytokine production. Data on plasma TT- and PPD-specific antibodies were obtained from 348 samples.

Several characteristics of participants for whom we obtained data on cytokine and/or antibody responses differed between urban and rural settings (Table 1). The prevalence of helminths, *P. falciparum* positivity (blood smear) and HIV was higher in the rural compared to the urban setting. Rural participants were significantly more likely to report anthelminthic or malaria treatment in the last year compared to urban participants. There were no urban-rural differences in sex distribution, but rural participants were somewhat younger on average than urban participants. Over 90% of participants in both settings reported and/or showed evidence of immunisation with BCG or tetanus in infancy, with marginally higher prevalence of any immunisation among urban participants. Urban participants were significantly more likely to have a BCG scar compared to rural participants (Table 1).

**Table 1:** Participants' characteristics.

Characteristic	Urban	Rural	P value*
	**n/N**^¶^**(%)**^#^	**n/N**^¶^**(%)**^#^	
Male sex	161/350 (46.0)	500/966 (49.3)	0.293
Age			
1–4	116/350 (33.1)	404/966 (40.5)	
5–8	87/350 (24.9)	299/966 (29.0)	
9–12	64/350 (18.3)	159/966 (17.2)	
13–17	83/350 (23.7)	104/966 (13.4)	**0.026**
Place of birth			
City	17/345 (4.9)	11/963 (1.5)	
Town	302/345 (87.5)	56/963 (5.6)	
Village	26/345 (7.5)	896/963 (92.9)	**<0.001**
Occupation			
Student or child	329/345 (95.4)	937/963 (97.1)	
Unemployed or house wife	3/345 (0.9)	7/963 (0.9)	
Agricultural, fishing or lake related	2/345 (0.6)	13/963 (1.0)	
Professional or service providers (Shops, saloons, bars, restaurants, entertainment)	11/345 (3.2)	6/963 (0.9)	**0.042**
Ever received immunisation			
Any	338/346 (97.7)	619/662 (93.3)	**0.047**
Bacille Calmette–Guérin (BCG)	315/336 (93.8)	586/619 (92.6)	0.757
Diphtheria, Pertussis and Tetanus (DPT)	313/336 (93.2)	571/619 (90.1)	0.420
Source of information about immunisation			
Health card	95/346 (27.5)	217/623 (32.5)	
Self/parent/guardian reported	242/346 (69.9)	401/623 (66.0)	
No information	9/346 (2.6)	5/623 (1.6)	0.509
Presence of BCG scar	263/350 (75.1)	645/955 (66.3)	**0.008**
Helminth infections			
*S. mansoni* (KK)	27/282 (9.6)	285/834 (35.4)	**<0.001**
*S. mansoni* intensity (KK)			
Uninfected	255/282 (90.4)	549/834 (64.7)	
Light	13/282 (4.6)	140/834 (17.1)	
Moderate	11/282 (3.9)	80/834 (10.2)	
Heavy	3/282 (1.1)	65/834 (8.1)	**<0.001**
*S. mansoni* (PCR)	46/277 (16.6)	417/830 (51.9)	**<0.001**
Any nematode^¥^	18/276 (8.0)	179/830 (23.3)	**<0.001**
*A. lumbricoides* (KK)	0/282 (0.0)	8/834 (0.7)	0.349
*N. americanus* (KK and/or PCR)	9/276 (3.3)	62/830 (7.2)	**0.031**
*T. trichiura* (KK)	7/282 (2.5)	112/834 (11.9)	**0.003**
*S. stercoralis* (PCR)	3/277 (1.1)	20/830 (2.2)	0.289
Any anthelminthic treatment in last year	263/346 (76.0)	800/961 (81.4)	**0.026**
*P. falciparum* positivity (blood smear)	0/334 (0.0)	64/960 (7.2)	**<0.001**
Malaria treatment in the last year	147/344 (42.7)	710/963 (72.9)	**<0.001**
HIV infection	3/337 (0.9)	12/513 (2.8)	**0.019**

**KK**: Kato-Katz; **PCR**: Polymerase Chain Reaction; ^***¶***^denominators for each characteristic represent the number of participants that responded to the respective questions or for whom a sample was available for laboratory analysis; *P values obtained from Pearson chi square test, values in bold are significant at 0.05; ^**¥**^infection with any of *A. lumbricoides, N. americanus*, *T. trichiura*, or *S. stercoralis*; ^**#**^Percentages adjusted for survey design.

### 3.2 Urban-rural comparisons of PPD- and tetanus toxoid-specific responses

PPD-specific IFN-γ (*p* *<* *.001*) and IL-13 (*p* *<* *.001*), and TT-specific IL-13 (*p* *=* *.003*) and IgG concentrations (*p* *=* *.002*) were significantly lower among rural, compared to urban survey participants, after adjusting for age, sex, place of birth and BCG scar (Table 2). In contrast, PPD-specific IgE concentrations (*p* *=* *.022*) were significantly higher among rural participants.

**Table 2:** Purified protein derivative- and tetanus toxoid-specific responses: urban-rural comparisons.

Vaccine antigen	Cytokine / Antibody	Geometric mean^β^		Unadjusted		Adjusted for age, sex, BCG scar and place of birth	
				GMR (95% CI)^#^	P value^§^	GMR (95% CI)^#^	P value^§^
		Urban*	Rural				
PPD	IFN-γ	831.3	164.0	0.20 (0.13, 0.30)	**<0.001**	0.22 (0.14, 0.35)	**<0.001**
	IL-5	13.8	14.2	1.02 (0.67, 1.57)	0.917	1.04 (0.43, 2.52)	0.932
	IL-13	34.9	8.4	0.24 (0.17, 0.35)	**<0.001**	0.22 (0.12, 0.40)	**<0.001**
	IL-10	37.8	28.1	0.74 (0.52, 1.06)	0.098	0.55 (0.27, 1.10)	0.086
						
	IgG	19,700.9	22,549.0	1.15 (1.01, 1.30)	**0.033**	1.07 (0.94, 1.21)	0.295
	IgE	92.7	115.2	1.24 (0.09, 1.72)	0.182	1.56 (1.07, 2.29)	**0.022**
	IgG4	92.0	94.7	1.03 (1.00, 1.06)	**0.026**	1.02 (0.96, 1.09)	0.501
TT	IFN-γ	14.6	7.3	0.50 (0.24, 1.08)	0.077	0.69 (0.27, 1.78)	0.435
	IL-5	5.5	3.7	0.66 (0.36, 1.23)	0.185	0.64 (0.34, 1.19)	0.152
	IL-13	11.2	3.0	0.27 (0.13, 0.52)	**<0.001**	0.36 (0.19, 0.68)	**0.003**
	IL-10	5.3	5.8	1.07 (0.78, 1.47)	0.652	0.96 (0.63, 1.48)	0.859
	IgG	51,760.4	47,418.3	0.92 (0.84, 0.99)	**0.041**	0.83 (0.74, 0.93)	**0.002**
	IgE	504.7	679.0	1.35 (1.01, 1.79)	**0.042**	1.12 (0.53, 2.39)	0.758
	IgG4	14,006.7	12,289.7	0.88 (0.76, 1.02)	0.080	0.82 (0.64, 1.05)	0.109

*reference category is urban setting; ^**β**^Cytokine concentrations in pg/ml, antibody concentrations in ng/ml; ^**#**^Geometric mean ratios (GMR) and 95% CI adjusted for survey design; ^**§**^P values in bold are significant at 0.05; **PPD**: purified protein derivative; **TT**: tetanus toxoid; **95% CI**: 95% confidence interval.

### 3.3 PPD- and TT-specific responses, and associations with age and sex

**Fig. 2:**
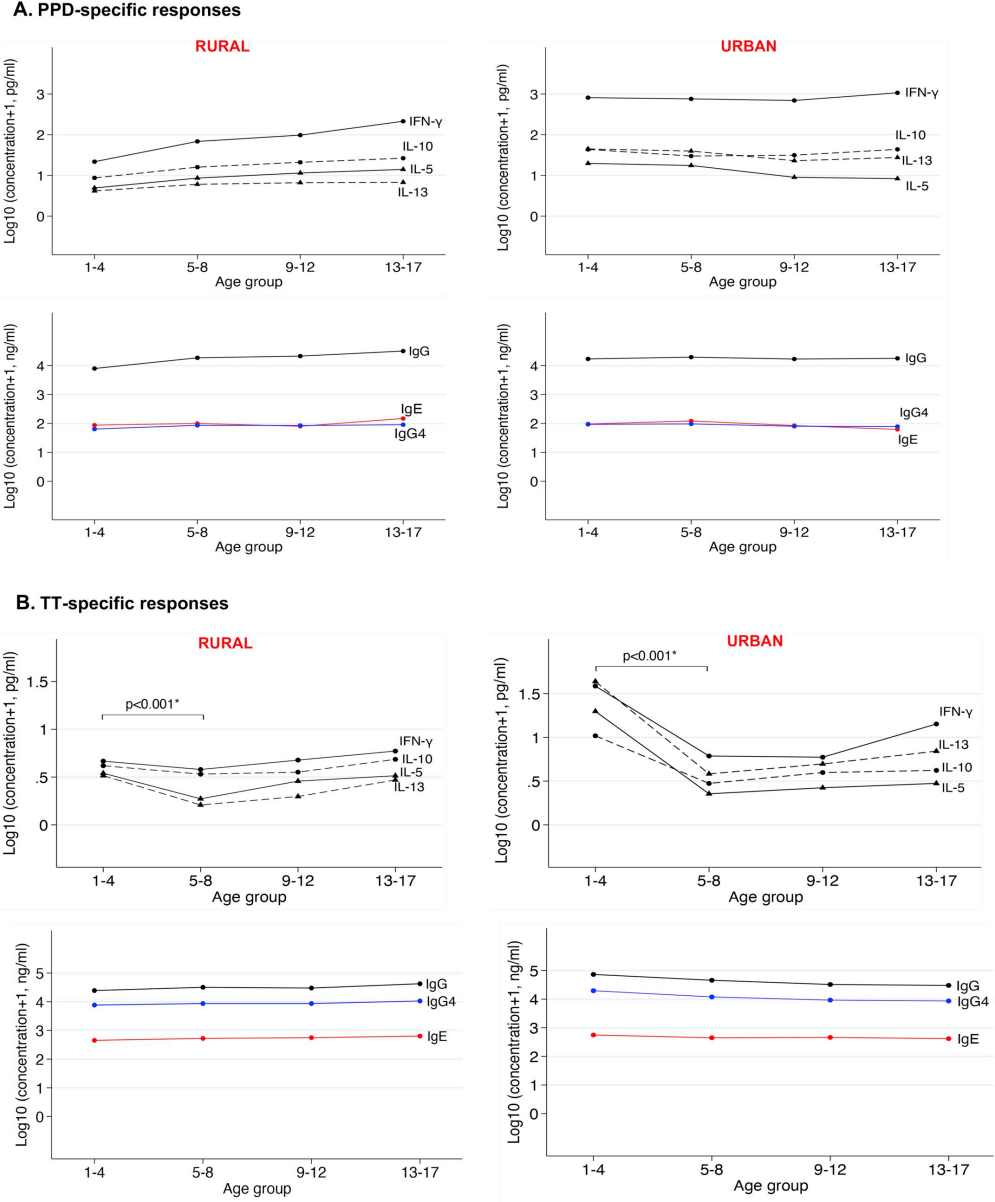
Age-stratified mean concentrations of PPD- and TT-specific cytokines and antibodies. A: Mean purified protein derivative (PPD)-specific antibody and cytokine responses stratified by age (in years) in the rural and urban surveys. B: Mean tetanus toxoid (TT)-specific antibody and cytokine responses stratified by age (in years) in the rural and urban surveys. Plotted results are from all participants, irrespective of *Schistosoma mansoni* infection status. *Mann-Whitney test was used to assess differences in cytokine responses between 1 and 4-year olds and 5–8-year olds. P < .001 for all four TT-specific cytokines.

In the rural survey, mean PPD-specific cytokines and PPD-specific IgG concentrations increased gradually with age; however, in the urban survey, age differences in PPD-specific cytokine and antibody responses were not apparent (Fig. 2). In both surveys, mean TT-specific cytokine concentrations were significantly lower in five- to eight-year olds compared to one- to four-year olds (*p* *<* *.001)*, after which they either plateaued or increased gradually with age.

There were no significant differences in PPD-and TT-specific responses between males and females in either the rural or the urban setting (data not shown).

### 3.4 Associations between current *Schistosoma mansoni* infection status and PPD- and tetanus toxoid-specific immune responses

Table 3 summarises associations between *Sm* infection (KK^+^ and/or PCR^+^) and responses to TT and PPD separately in each setting.

**Table 3:** Association between current *S. mansoni* infection status and PPD- and tetanus toxoid-specific responses in the rural and urban setting.

Vaccine antigen	Cytokine / Antibody	Geometric mean^β^		Unadjusted		Adjusted for age, sex, BCG scar and place of birth	
				GMR (95% CI)^#^	P value^§^	GMR (95% CI)^#^	P value^§^
A: Rural survey							
		*Sm*-*	***Sm+***				
PPD	IFN-γ	180.9	143.1	0.79 (0.57, 1.07)	0.123	0.70 (0.50, 0.99)	**0.043**
	IL-5	16.7	13.2	0.79 (0.48, 1.31)	0.350	0.72 (0.42, 1.25)	0.232
	IL-13	10.0	7.6	0.76 (0.51, 1.12)	0.161	0.75 (0.48, 1.19)	0.211
	IL-10	28.7	26.8	0.93 (0.61, 1.44)	0.752	0.98 (0.65, 1.47)	0.901
	IgG	20,696.3	24,511.4	1.18 (1.08, 1.30)	**0.001**	1.04 (0.96, 1.13)	0.315
	IgE	97.8	128.8	1.32 (0.89, 1.96)	0.166	1.40 (0.90, 2.18)	0.131
	IgG4	92.8	95.1	1.03 (0.99, 1.07)	0.197	1.03 (0.98, 1.07)	0.286
TT	IFN-γ	7.2	6.6	0.92 (0.47, 1.79)	0.804	0.94 (0.51, 1.71)	0.821
	IL-5	4.1	3.1	0.76 (0.49, 1.20)	0.238	0.83 (0.49, 1.40)	0.472
	IL-13	3.3	2.6	0.80 (0.54, 1.17)	0.241	0.86 (0.57, 1.30)	0.465
	IL-10	5.9	5.1	0.86 (0.61, 1.22)	0.407	0.98 (0.67, 1.43)	0.916
	IgG	49,742.5	44,871.3	0.90 (0.82, 0.99)	**0.036**	0.95 (0.86, 1.04)	0.272
	IgE	702.1	637.5	0.91 (0.58, 1.42)	0.662	0.96 (0.60, 1.55)	0.873
	IgG4	11,763.9	12,533.5	1.07 (0.91, 1.25)	0.431	1.17 (0.97, 1.41)	0.095

B: Urban survey							
PPD	IFN-γ	787.0	915.8	1.16 (0.78, 1.73)	0.430	1.02 (0.72, 1.43)	0.916
	IL-5	13.4	14.4	1.08 (0.44, 2.64)	0.861	1.21 (0.63, 2.34)	0.545
	IL-13	31.0	49.5	1.60 (0.94, 2.71)	0.080	1.75 (1.19, 2.56)	**0.007**
	IL-10	38.6	38.3	0.99 (0.62, 1.59)	0.977	0.88 (0.55, 1.41)	0.574
	IgG	18,794.9	24,511.1	1.30 (1.14, 1.49)	**<0.001**	1.14 (1.00, 1.31)	0.054
	IgE	93.7	107.3	1.15 (0.66, 2.00)	0.615	1.20 (0.70, 2.06)	0.497
	IgG4	91.3	101.3	1.11 (0.99, 1.24)	0.065	1.12 (1.00, 1.26)	**0.046**
TT	IFN-γ	15.9	4.6	0.29 (0.11, 0.76)	**0.015**	0.35 (0.13, 0.91)	**0.033**
	IL-5	5.5	2.7	0.49 (0.23, 1.06)	0.068	0.79 (0.37, 1.69)	0.519
	IL-13	11.9	4.0	0.33 (0.16, 0.71)	**0.007**	0.53 (0.24, 1.20)	0.117
	IL-10	5.4	4.6	0.85 (0.55, 1.32)	0.451	0.98 (0.62, 1.53)	0.907
	IgG	51,109.5	41,861.6	0.82 (0.71, 0.95)	**0.011**	0.92 (0.79, 1.07)	0.257
	IgE	493.3	353.4	0.72 (0.20, 2.52)	0.586	0.73 (0.23, 2.26)	0.560
	IgG4	13,176.7	12,644.4	0.96 (0.67, 1.38)	0.814	1.13 (0.84, 1.52)	0.399

**Sm+**: positive Kato-Katz and/or PCR test for diagnosis of current infection with *Schistosoma mansoni;***Sm-**: negative Kato-Katz and PCR test for diagnosis of current infection with *Schistosoma mansoni;* *reference category is *Schistosoma mansoni* uninfected group; ^**β**^Cytokine concentrations in pg/ml, antibody concentrations in ng/ml; ^**#**^Geometric mean ratios (GMR) and 95% CI adjusted for survey design; ^**§**^P values in bold are significant at 0.05; **PPD**: purified protein derivative; **TT**: tetanus toxoid; **95% CI**: 95% confidence interval.

In the rural survey, there was a weak inverse association between *Sm* infection and PPD-specific IFN-γ (*adjusted GMR [95% CI]: 0.70 [0.50, 0.99], p* *=* *.043*); however, there were no significant age- and sex-adjusted associations with any other PPD- or TT-specific cytokine or antibody responses (Table 3A). We did not observe a dose-response relationship with *Sm* infection intensity (supplementary Table S1).

In the urban survey, the picture was more complex. There were significant positive associations between *Sm* infection and PPD-specific cytokine and antibody responses, as observed for PPD-specific IL-13 (*aGMR 1.75 [1.19, 2.56], p* *=* *.007*) and IgG4 responses (*aGMR 1.12 [1.00, 1.26], p* *=* *.046*) [Table 3B]. Conversely, for associations between *Sm* and TT-specific responses, geometric mean concentrations of cytokine and antibody responses were lower among infected participants, although only the association with TT-specific IFN-γ reached statistical significance (*aGMR 0.35 [0.13,0.91], p* *=* *.033)*.

### 3.5 Associations between current nematode infection status and PPD- and tetanus toxoid-specific immune responses

Prevalence of infection with each of the nematodes *A. lumbricoides, N. americanus*, *T. trichiura*, and *S. stercoralis* was low; therefore, they were grouped to form one variable representing infection with any nematode. Table 4A summarises associations between ‘any nematode’ infection and responses to TT and PPD in the rural survey. Infection with ‘any nematode’ was positively associated with PPD-specific IgG (*aGMR 1.10 [1.02, 1.19], p* *=* *.013*) but not with any other PPD- or TT-specific responses.

**Table 4:** Association between current infection with any nematode and PPD- and tetanus toxoid-specific responses in the rural and urban setting.

Vaccine antigen	Cytokine / Antibody	Geometric mean^β^		Unadjusted		Adjusted for age, sex, BCG scar and place of birth	
				GMR (95% CI)^#^	P value^§^	GMR (95% CI)^#^	P value^§^
A: Rural survey							
		Nm-*	**Nm*+***				
PPD	IFN-γ	159.3	149.5	0.94 (0.61, 1.44)	0.761	0.81 (0.46, 1.42)	0.445
	IL-5	14.5	14.8	1.02 (0.74, 1.41)	0.897	0.92 (0.63, 1.36)	0.661
	IL-13	8.5	8.4	0.98 (0.75, 1.28)	0.887	0.90 (0.61, 1.34)	0.597
	IL-10	27.5	27.4	1.00 (0.78, 1.27)	0.967	1.00 (0.76, 1.32)	0.998
	IgG	21,838.4	26,564.9	1.22 (1.12, 1.32)	**<0.001**	1.10 (1.02, 1.19)	**0.013**
	IgE	112.3	119.2	1.06 (0.77, 1.47)	0.709	1.08 (0.83, 1.41)	0.558
	IgG4	92.9	98.8	1.06 (1.00, 1.13)	**0.037**	1.06 (1.00, 1.13)	0.065
TT	IFN-γ	6.8	6.9	1.00(0.53, 1.89)	0.994	0.93 (0.57, 1.53)	0.777
	IL-5	3.8	2.6	0.70 (0.48, 1.02)	0.065	0.73 (0.53, 1.02)	0.064
	IL-13	3.0	2.4	0.81(0.60, 1.11)	0.178	0.83 (0.62, 1.12)	0.209
	IL-10	5.7	4.5	0.78 (0.59, 1.03)	0.076	0.88 (0.70, 1.09)	0.242
	IgG	47,359.2	45,680.6	0.97 (0.87, 1.07)	0.480	0.98 (0.89, 1.08)	0.716
	IgE	655.6	725.4	1.11 (0.73, 1.68)	0.622	1.18 (0.80, 1.76)	0.389
	IgG4	12,001.9	12,923.3	1.08 (0.82, 1.41)	0.577	1.11 (0.86, 1.43)	0.405

B: Urban survey							
PPD	IFN-γ	790.4	1163.6	1.47 (0.96, 2.26)	0.074	1.39 (0.92, 2.10)	0.109
	IL-5	13.3	17.8	1.34 (0.36, 5.01)	0.649	1.52 (0.46, 5.03)	0.467
	IL-13	33.6	37.2	1.11 (0.42, 2.89)	0.826	1.21 (0.53, 2.78)	0.637
	IL-10	37.9	49.3	1.30 (0.90, 1.87)	0.146	1.27 (0.84, 1.92)	0.235
	IgG	19,452.7	22,875.3	1.18 (1.05, 1.32)	**0.008**	1.05 (0.93, 1.19)	0.409
	IgE	97.1	79.0	0.81 (0.39, 1.72)	0.572	0.78 (0.33, 1.86)	0.561
	IgG4	92.3	102.5	1.11 (0.88, 1.40)	0.362	1.12 (0.89, 1.41)	0.315
TT	IFN-γ	13.1	7.4	0.56 (0.21, 1.53)	0.240	0.68 (0.29, 1.57)	0.337
	IL-5	4.8	4.4	0.92 (0.20, 4.30)	0.913	1.25 (0.32, 4.88)	0.735
	IL-13	10.2	4.6	0.46 (0.09, 2.37)	0.327	0.62 (0.14, 2.86)	0.518
	IL-10	5.2	4.5	0.86 (0.36, 2.08)	0.722	0.95 (0.43, 2.10)	0.888
	IgG	50,320.9	37,708.1	0.75 (0.64, 0.88)	**0.001**	0.84 (0.71, 0.99)	**0.036**
	IgE	469.7	421.3	0.90 (0.16, 4.96)	0.896	1.02 (0.19, 5.48)	0.979
	IgG4	13,485.6	8291.7	0.62 (0.50, 0.76)	**<0.001**	0.68 (0.53, 0.86)	**0.003**

**Nm+**: positive Kato-Katz or PCR test for diagnosis of current infection with any of *A. lumbricoides, N. americanus*, *T. trichiura*, or *S. stercoralis;***Nm-**: negative Kato-Katz and PCR test for diagnosis of current infection with any of *A. lumbricoides, N. americanus*, *T. trichiura*, or *S. stercoralis;* *reference category is uninfected group; ^**β**^Cytokine concentrations in pg/ml, antibody concentrations in ng/ml; ^**#**^Geometric mean ratios (GMR) and 95% CI adjusted for survey design; ^**§**^P values in bold are significant at 0.05; **PPD**: purified protein derivative; **TT**: tetanus toxoid; **95% CI**: 95% confidence interval.

In the urban survey, infection with ‘any nematode’ was inversely associated with TT-specific IgG (*aGMR 0.84 [0.71, 0.99], p* *=* *.036*) and IgG4 (*aGMR 0.68 [0.53, 0.86], p* *=* *.003*) [Table 4B]. However, there were no significant associations between nematode infections and PPD-specific cytokine and antibody responses or TT-specific cytokines.

### 3.6 Urban-rural differences in PPD- and tetanus toxoid-specific immune responses: A role for helminths?

To assess for any potential role of helminth infections in urban-rural differences in PPD- and TT-specific responses, we conducted additional adjustment for current infection with *Sm* or any nematode (Table 5). For PPD-specific IgE and for TT-specific IL-13 and IgG responses, modest differences in GMRs before and after adjustment were observed. However, for the rest of the responses, there were only very small changes in GMRs before and after adjustment for helminth infections.

**Table 5:** Do helminths underpin urban-rural differences in PPD- and tetanus toxoid-specific immune responses?

				Adjusted for age, sex, BCG scar and place of birth				Adjusted for age, sex, BCG scar, place of birth **and *S. mansoni* infection**			Adjusted for age, sex, BCG scar, place of birth **and any** **nematode infection**^**¶**^	
Vaccine antigen	Cytokine / Antibody	Geometric mean^β^			GMR (95% CI)^#^	P value^§^		GMR (95% CI)^#^	P value^§^		GMR (95% CI)^#^	P value^§^
		Urban*	**Rural**									
PPD	IFN-γ	831.3	164.0		0.22 (0.14, 0.35)	**<0.001**		0.24 (0.14, 0.41)	**<0.001**		0.22 (0.13, 0.37)	**<0.001**
	IL-5	13.8	14.2		1.04 (0.43, 2.52)	0.932		1.26 (0.59, 2.68)	0.542		1.16 (0.50, 2.70)	0.727
	IL-13	34.9	8.4		0.22 (0.12, 0.40)	**<0.001**		0.24 (0.13, 0.43)	**<0.001**		0.23 (0.12, 0.42)	**<0.001**
	IL-10	37.8	28.1		0.55 (0.27, 1.10)	0.086		0.54 (0.26, 1.13)	0.099		0.54 (0.24, 1.22)	0.135
									
	IgG	19,700.9	22,549.0		1.07 (0.94, 1.21)	0.295		1.05 (0.911, 1.20)	0.523		1.05 (0.91, 1.21)	0.470
	IgE	92.7	115.2		1.56 (1.07, 2.29)	**0.022**		1.24 (0.81, 1.88)	0.320		1.37 (0.89, 2.11)	0.147
	IgG4	92.0	94.7		1.02 (0.96, 1.09)	0.501		0.99 (0.92, 1.06)	0.673		0.99 (0.93, 1.06)	0.836
									
TT	IFN-γ	14.6	7.3		0.69 (0.27, 1.78)	0.435		0.84 (0.34, 2.06)	0.694		0.79 (0.31, 1.99)	0.608
	IL-5	5.5	3.7		0.64 (0.34, 1.19)	0.152		0.87 (0.49, 1.56)	0.641		0.85 (0.49, 1.46)	0.539
	IL-13	11.2	3.0		0.36 (0.19, 0.68)	**0.003**		0.48 (0.26, 0.90)	**0.023**		0.46 (0.24, 0.87)	**0.018**
	IL-10	5.3	5.8		0.96 (0.63, 1.48)	0.859		0.96 (0.56, 1.66)	0.881		0.97 (0.59, 1.61)	0.918
									
	IgG	51,760.4	47,418.3		0.83 (0.74, 0.93)	**0.002**		0.86 (0.75, 0.98)	**0.024**		0.85 (0.74, 0.96)	**0.013**
	IgE	504.7	679.0		1.12 (0.53, 2.39)	0.758		1.15 (0.49, 2.72)	0.740		1.10 (0.44, 2.74)	0.827
	IgG4	14,006.7	12,289.7		0.82 (0.64, 1.05)	0.109		0.82 (0.62, 1.08)	0.148		0.86 (0.66, 1.11)	0.239

*reference category is urban setting; ^**β**^Cytokine concentrations in pg/ml, antibody concentrations in ng/ml; ^**#**^Geometric mean ratios (GMR) and 95% CI adjusted for survey design; ^**¶**^Infection with any of *A. lumbricoides, N. americanus*, *T. trichiura*, or *S. stercoralis*; ^**§**^P values in bold are significant at 0.05; **PPD**: purified protein derivative; **TT**: tetanus toxoid; **95% CI**: 95% confidence interval.

## 4 Discussion

In rural helminth-endemic Ugandan fishing villages and nearby urban communities with lower helminth infection intensity, we examined cytokine and antibody responses to purified protein derivative and tetanus toxoid, and show that these responses differ between urban and rural populations. We hypothesised that adjusting for current helminth infections would abrogate statistically significant urban-rural differences in PPD- and TT-specific concentrations; however, changes in results after adjustment were, at most, modest. Associations between PPD- / TT-specific concentrations and *Sm* and nematode infections did not follow a consistent pattern between settings, or types of infection: both inverse and positive associations were observed.

Rural participants resided in a high helminth exposure setting; prevalence of infection with *Sm* in this group is likely to be much higher than shown here by the Kato-Katz and PCR tests: prevalence by the urine circulating cathodic antigen (CCA) surpasses 80% [26]. Therefore, a considerable number of Kato-Katz and PCR negative participants were probably lightly infected, with Kato-Katz / PCR positivity being mostly indicative of moderate-to-heavy *Sm* infection. The urban setting also had a considerable prevalence of light helminth infections. Nonetheless, the two settings are still relatively dissimilar, as helminth infection prevalence is significantly higher in the rural compared to the urban setting, providing an important opportunity for exploring the role of helminths in urban-rural differences in response to vaccine antigens.

Responses to PPD and TT are herein taken to represent recall immune responses to BCG and tetanus vaccination in infancy. Vaccine responses may wane over time; however, PPD- or TT-specific concentrations in our study were either generally similar across age groups or appeared to increase gradually with age. The exception was TT-specific cytokine responses, which were significantly higher in one- to four-year olds compared to five- to eight-year olds, after which they either plateaued or increased gradually with age, perhaps due to immunisations given later in life. It is also plausible that the gradual increase with age is due to continuous exposure to environmental antigens that are cross-reactive with vaccine antigens. Our questionnaires enabled collection of data on history of immunisations; there were no substantive differences between comparison groups. However, BCG scar prevalence was higher among urban compared to rural participants. Differences in scar prevalence might reflect differences in immunological response to BCG vaccination, probably influenced by differential environmental sensitisation between the two settings. Of note, our findings were similar regardless of whether or not BCG scar was adjusted for in the analysis. It is also important to note that the vaccines were not administered by our study team; therefore, some of the observed differences may be based on differential vaccination provision/uptake.

Mutual inhibition of Th1- and Th2-type immune responses and the distinct ability of several vaccine targets and helminth antigens to induce Th1 and Th2 responses [35], respectively, initially underlay the hypothesis that helminths may impair vaccine responses. The inverse association between *Sm* infection and PPD-specific IFN-γ among rural survey participants is consistent with this hypothesis. Our observations of inverse associations between ‘any nematode’ infection and TT-specific IgG and IgG4 in the urban survey further support a role for bystander effects of helminth-mediated immunoregulation [36].

We observed positive associations between *Sm* infection and PPD-specific IL-13, and IgG4 in the urban survey, and between nematode infection and PPD-specific IgG in the rural survey. There have been observations from other human and animal studies suggesting that in some instances specific helminth antigens may enhance or bias vaccine-specific responses to a particular immune phenotype. A study in Ecuador showed a positive association between maternal geohelminth infection and infant IgA responses to oral polio vaccine and rotavirus vaccine [37], while in Uganda, maternal Strongyloidiasis was associated with enhanced responses to pertussis toxin, *Haemophilus influenzae* B and hepatitis B vaccine antigens in infancy [38]. In mice, the *Onchocerca volvulus* activation-associated secreted protein (*Ov-*ASP-1) was shown to boost Th1-biased cellular and antibody reactivity to influenza vaccines [39,40], and early studies in mice suggested that *Trichinella spiralis* infection potentiated cellular immune responses to BCG [41,42].

As discussed in our review [19] effects of helminth infections on vaccine responses are complex. Helminth species, life stage and exposure timing and intensity may influence vaccine response. The type and characteristics of the vaccine may also determine whether helminth infections will have suppressive or enhancing effects: live and non-live, oral and parenteral, priming and boosting vaccines may be affected differently. Furthermore, helminths interact extensively with gut microbiota [43], with possible long-term immunological consequences [44,45], including modulation of vaccine-specific responses [46]. All these factors should be considered in large immunoepidemiological studies investigating the role of environmental sensitisation on vaccine-specific immune responses and efficacy.

We conducted many statistical tests, but did not adjust for multiplicity. However, we focus on patterns of associations and on biological credibility of results based on other published works. Our observations of urban-rural differences are consistent with previous studies [[10], [11], [12], [13]], especially with regard to lower concentrations of PPD- and TT-specific cytokines and antibodies in the rural compared to urban survey. Our rural setting has a significantly higher prevalence of *Sm* and geohelminth infections compared to the urban setting. The implication of this for the urban-rural differences in vaccine response was inconclusive from our data; helminths likely work in concert with other environmental exposures to influence vaccine response. Further studies designed specifically to examine effects of environmental and parasite exposures on vaccine efficacy are warranted.

## Funding

The LaVIISWA study (the ‘rural survey’) and the urban survey were funded by the Wellcome Trust, grant 095778 awarded to AME. GN is an honorary member, and RES a PhD fellow, of the Makerere University – Uganda Virus Research Institute Centre of Excellence for Infection and Immunity Research and Training (MUII-plus). MUII-plus is funded under the DELTAS Africa Initiative. The DELTAS Africa Initiative is an independent funding scheme of the 10.13039/501100011858African Academy of Sciences (AAS), 10.13039/501100014163Alliance for Accelerating Excellence in Science in Africa (AESA) and supported by the New Partnership for Africa's Development Planning and Coordinating Agency (NEPAD Agency) with funding from the 10.13039/100010269Wellcome Trust (grant 107743) and the 10.13039/100013986UK Government. The MRC/UVRI and LSHTM Uganda Research Unit is jointly funded by the UK 10.13039/501100000265Medical Research Council (MRC) and the UK 10.13039/501100000278Department for International Development (DFID) under the MRC/DFID Concordat agreement and is also part of the EDCTP2 programme supported by the 10.13039/501100000780European Union.

## Author contributions

AME, GN, ELW and JK contributed to the conception and experimental design of the study. JK, GN and JN conducted the immunology experiments. RES and MN led and participated in the field and clinic procedures. AN, JK, GN and ELW analysed the data. GN, AN and JK wrote the manuscript, with all authors contributing to the interpretation of the results, and revision and approval of the final manuscript.

## Research data

The datasets generated during and/or analysed during the current study are available from the corresponding author on reasonable request.

## Declaration of Competing Interest

None.

## Appendix A Supplementary data

Supplementary material Image 1
