# Supplementary material for: Urban-rural differences in immune responses to mycobacterial and tetanus vaccine antigens in a tropical setting: A role for helminths?
Source: Parasitol Int. 2020 Oct;78:102132. doi: 10.1016/j.parint.2020.102132 (PMC7397513; doi:10.1016/j.parint.2020.102132)
Supplement: Supplementary file 1 — Supplementary material [file mmc1.docx]

**Urban-rural differences in immune responses to mycobacterial and tetanus vaccine antigens in a tropical setting: a role for helminths?**

**SUPPLEMENTARY INFORMATION**

**Supplementary methods**

*TT- and PPD-specific IgE ELISA.*

All but the first 2 columns of MICROLON® high binding 96-well plates (Greiner bio-one, UK) were coated with 50 μl of TT or PPD (both from Statens Serum Institut, Denmark) at a concentration of 5 μg/ml in bicarbonate (Na_2_CO_3_ + NaHCO_3_) buffer (0.1M, pH 9.6). Two-fold dilutions of human IgE (Calbiochem, Beeston, UK) standard, diluted in bicarbonate buffer, were added to the first 2 columns of each plate to form standard curves. The plates were then incubated overnight at 4ºC. Plates were washed with phosphate-buffered saline (PBS)-Tween 20 solution, blocked with 150 μl of 1% w/v skimmed milk diluted in PBS-Tween 20 at room temperature (RT), washed again and incubated overnight at 4ºC with 50 μl of plasma samples diluted 1/20 (TT assay), or 1/5 (PPD assay) with 0.1% w/v dried milk powder in PBS + 0.05% Tween 20 (assay buffer). Plates were washed and antibody binding detected by incubating the plates overnight at 4ºC with 0.5 μg/ml of biotinylated monoclonal mouse anti-human IgE (BD Pharmingen™). After washing, the plates were incubated for 1 hour with a streptavidin-horseradish peroxidase (strep-HRP) conjugate (Mast Group Ltd, Bootle, UK), diluted 1/3000 with assay buffer, at RT. Plates were washed again and developed by addition of 100 μl of o-phenylenediamine (Sigma-Aldrich) and reactions stopped with 30 μl of 2M Sulphuric acid. Optical density (OD) values were measured at 490 nm (reference wavelength 630 nm) on a 96-well plate ELISA reader. Plasma IgE concentrations (ng/ml) were interpolated from standard curves (generated after plotting known concentrations of serially-diluted IgE standards against their measured ODs) using a five-parameter curve fit using Gen5 data collection and analysis software (BioTek Instruments Inc, Vermont, Winooski, USA).

*TT- and PPD-specific IgG4 ELISA*

All but the first 2 columns of MICROLON® high binding 96-well plates (Greiner bio-one, UK) were coated with 50 μl of TT or PPD (both from Statens Serum Institut, Denmark) at a concentration of 5 μg/ml in bicarbonate (Na_2_CO_3_ + NaHCO_3_) buffer (0.1M, pH 9.6). Two-fold dilutions of human IgG4 (Sigma-Aldrich) standard, diluted in bicarbonate buffer, were added to the first 2 columns of each plate to form standard curves. The plates were then incubated overnight at 4ºC. Plates were washed with phosphate-buffered saline (PBS)-Tween 20 solution, blocked with 150 μl of 1% w/v skimmed milk diluted in PBS-Tween 20 at room temperature (RT), and incubated overnight at 4ºC with 50 μl of plasma samples diluted 1/300 (TT assay), or 1/5 (PPD assay) with 0.1% w/v dried milk powder in PBS + 0.05% Tween 20 (assay buffer). Plates were washed and antibody binding detected by incubating the plates overnight at 4ºC with 0.5 μg/ml of biotinylated monoclonal mouse anti-human IgG4 (BD Pharmingen™), followed by a one-hour incubation with a streptavidin-horseradish peroxidase (strep-HRP) conjugate (Mast Group Ltd, Bootle, UK), diluted 1/3000 with assay buffer, at RT. Plates were developed by addition of 100 μl of o-phenylenediamine (Sigma-Aldrich) and reactions stopped with 30 μl of 2M Sulphuric acid. Optical density (OD) values were measured at 490 nm (reference wavelength 630 nm) on a 96-well plate ELISA reader. Plasma IgG4 concentrations (ng/ml) were interpolated from standard curves (generated after plotting known concentrations of serially-diluted IgG4 standards against their measured ODs) using a five-parameter curve fit using Gen5 data collection and analysis software (BioTek Instruments Inc, Vermont, Winooski, USA).

*TT- and PPD-specific IgG ELISA*

All but the first 2 columns of MICROLON® high binding 96-well plates (Greiner bio-one, UK) were coated with 50 μl of TT or PPD (both from Statens Serum Institut, Denmark) at a concentration of 5 μg/ml in bicarbonate (Na_2_CO_3_ + NaHCO_3_) buffer (0.1M, pH 9.6). Two-fold dilutions of human IgG (Calbiochem, Beeston, UK) standard, diluted in bicarbonate buffer, were added to the first 2 columns of each plate to form standard curves. The plates were then incubated overnight at 4ºC. Plates were washed with phosphate-buffered saline (PBS)-Tween 20 solution, blocked with 150μl of 1% skimmed milk diluted in PBS-Tween 20 at room temperature (RT), and incubated overnight at 4ºC with 50μl of plasma samples diluted 1/100 with 0.1% skimmed milk in PBS-Tween 20 (assay buffer). Plates were washed and antibody binding detected by incubating the plates for 1 hour at RT with 0.5μg/ml of polyclonal rabbit anti-human IgG/HRP (Dako, Denmark). Plates were washed and then developed by addition of 100μl of o-phenylenediamine (Sigma-Aldrich) and reactions stopped after 20 minutes with 25μl of 2M Sulphuric acid. Optical density (OD) values were measured at 490nm (reference wavelength 630nm) on a 96-well plate ELISA reader. Plasma IgG concentrations (ng/ml) were interpolated from standard curves (generated after plotting known concentrations of serially-diluted IgG standards against their measured ODs) using a five-parameter curve fit using Gen5 data collection and analysis software (BioTek Instruments Inc, Vermont, Winooski, USA).

**Supplementary Table S1.** Association between *S. mansoni* infection intensity and PPD- and tetanus toxoid-specific responses in the rural setting

| **Vaccine antigen** | **Cytokine / Antibody** | **Geometric mean^β^** | | | |  | **GMR (95% CI)^#^** | | |
| --- | --- | --- | --- | --- | --- | --- | --- | --- | --- |
|  |  | SmKK- | SmKK^low^ | SmKK^mod^ | SmKK^heavy^ |  | SmKK^low^ | SmKK^mod^ | SmKK^heavy^ |
| PPD | IFN-γ | 188.2 | **110.5** | 130.4 | 128.5 |  | **0.54 (0.33, 0.89)** | 0.68 (0.29, 1.63) | 0.57 (0.24, 1.37) |
|  | IL-5 | 14.5 | 13.9 | 12.5 | 19.0 |  | 0.90 (0.43, 1.89) | 0.86 (0.30, 2.42) | 1.14 (0.62, 2.09) |
|  | IL-13 | 8.9 | 6.6 | 8.7 | 9.9 |  | 0.78 (0.46, 1.32) | 1.04 (0.41, 2.66) | 1.19 (0.69, 2.06) |
|  | IL-10 | 26.7 | 28.9 | 26.9 | 31.6 |  | 1.12 (0.85, 1.47) | 1.13 (0.63, 2.02) | 1.31 (0.83, 2.09) |
|  |  |  |  |  |  |  |  |  |  |
|  | IgG | 21096.7 | 24464.9 | 26257.5 | 28431.4 |  | 1.05 (0.92, 1.20) | 1.08 (0.87, 1.33) | 1.10 (0.95, 1.28) |
|  | IgE | 107.3 | 121.1 | 112.1 | 142.7 |  | 1.20 (0.80, 1.79) | 1.15 (0.67, 1.98) | 1.37 (0.59, 3.19) |
|  | IgG4 | 93.1 | 90.7 | 95.8 | **108.0** |  | 0.97 (0.92, 1.03) | 1.02 (0.98, 1.06) | **1.17 (1.05, 1.29)** |
|  |  |  |  |  |  |  |  |  |  |
| TT | IFN-γ | 7.2 | 7.7 | 7.0 | 3.8 |  | 1.08 (0.46, 2.54) | 0.94 (0.35, 2.54) | 0.57 (0.23, 1.38) |
|  | IL-5 | 3.6 | 3.4 | 2.4 | 4.6 |  | 1.00 (0.62, 1.62) | 0.77 (0.47, 1.26) | 1.61 (0.55, 4.74) |
|  | IL-13 | 3.1 | 2.5 | 2.1 | 2.8 |  | 0.86 (0.54, 1.39) | 0.75 (0.53, 1.06) | 1.11 (0.56, 2.17) |
|  | IL-10 | 6.0 | 5.1 | 3.9 | 5.4 |  | 0.95 (0.62, 1.45) | 0.85 (0.45, 1.60) | 1.13 (0.53, 2.37) |
|  |  |  |  |  |  |  |  |  |  |
|  | IgG | 49033.1 | 45348.8 | 43282.4 | 40727.4 |  | 0.97 (0.87, 1.09) | 0.96 (0.87, 1.06) | 0.92 (0.81, 1.04) |
|  | IgE | 616.2 | 713.3 | 750.3 | 954.9 |  | 1.33 (0.57, 3.10) | 1.50 (0.58, 3.85) | 1.74 (0.89, 3.39) |
|  | IgG4 | 12259.9 | 11877.2 | 13031.3 | 11678.8 |  | 1.06 (0.84, 1.33) | 1.20 (0.98, 1.48) | 1.09 (0.72, 1.64) |

**SmKK-**: Kato-Katz negative result (*S. mansoni*), single stool sample; **SmKK^low^**: Kato-Katz positivity, low infection intensity (1-99 eggs/g); **SmKK^mod^**: Kato-Katz positivity, moderate infection intensity (100-399 eggs/g); **SmKK^heavy^**: Kato-Katz positivity, heavy infection intensity (≥400 eggs/g)

*reference category is *Schistosoma mansoni* uninfected group

**^β^**Cytokine concentrations in pg/ml, antibody concentrations in ng/ml

**^#^**Geometric mean ratios (GMR) and 95% CI adjusted for survey design, age, sex, BCG scar and place of birth.

**^§^**P values in bold are significant at 0.05

**PPD**: purified protein derivative

**TT**: tetanus toxoid

**95% CI**: 95% confidence interval
